# Maternal prepregnancy obesity and gestational diabetes influence on adverse perinatal outcomes

**DOI:** 10.20945/2359-3997000000605

**Published:** 2023-05-10

**Authors:** Leonardo Souza de Carvalho, Antônia Aparecida Deluca de Oliveira, Tassiana Cristina Martins Grabovski, Carla Christina Renzo, Rodrigo Ribeiro e Silva, Sabrina Hafemann Loz, Jean Carl Silva

**Affiliations:** 1 Universidade da Região de Joinville Programa de Mestrado em Saúde e Meio Ambiente Joinville SC Brasil Programa de Mestrado em Saúde e Meio Ambiente, Universidade da Região de Joinville (Univille), Joinville, SC, Brasil; 2 Universidade da Região de Joinville Faculdade de Medicina Joinville SC Brasil Faculdade de Medicina, Universidade da Região de Joinville (Univille), Joinville, SC, Brasil.; 3 Maternidade Darcy Vargas Joinville SC Brasil Maternidade Darcy Vargas, Joinville, SC, Brasil

**Keywords:** Prepregnancy obesity, gestational diabetes, perinatal outcome

## Abstract

**Objective:**

Evaluate the influence of isolated and associated prepregnancy obesity and gestational diabetes mellitus (GDM) on adverse perinatal outcomes.

**Materials and methods:**

Cross-sectional observational study with women who delivered at a Brazilian Maternity Hospital, between August and December 2020. Data were collected by interview with application form, and medical records. Sample was stratified by body mass index (BMI) and GDM screening in four groups: no obesity (BMI < 30 kg/m^2^) no GDM – reference; isolated GDM; isolated obesity (BMI ≥ 30 kg/m^2^); and obesity with GDM. Preeclampsia (PE), cesarean section (CS), large-for-gestational-age (LGA) newborn and admission to neonatal intensive care unit (NICU) were analyzed by odds ratio (OR) adjusted for confounding factors, adopting 95% confidence interval (CI) and *P* < 0.05 statistically significant.

**Results:**

From 1,618 participants, isolated obesity group (233/14.40%) had high chance of PE (OR = 2.16; CI: 1.364-3.426; P = 0.001), isolated GDM group (190/11.74%) had high chance of CS (OR = 1.736; CI: 1.136-2.652; P = 0.011) and NICU admission (OR = 2.32; CI: 1.265-4.261; *P* = 0.007), and obesity with GDM group (121/7.48%) had high chance of PE (OR = 1.93; CI: 1.074-3.484; *P* = 0.028), CS (OR = 1.925; CI: 1.124-3.298; *P* = 0.017) and LGA newborn (OR = 1.81; CI: 1.027-3.204; *P* = 0.040), compared with reference (1,074/66.38%).

**Conclusion:**

Obesity and GDM enhances the chance of different negative outcomes, worsening this prognosis when associated.

## INTRODUCTION

The World Health Organization (WHO) defined gestational diabetes mellitus (GDM) as hyperglycemia first detected during pregnancy, with glycemic levels that do not meet diagnostic cutoff values of diabetes mellitus (DM) ( 1 , 2 ). In the latest and more restricted criteria, the American Diabetes Association defined GDM as diabetes diagnosed in the second or third trimester of pregnancy that was not clearly overt diabetes prior to gestation ( 3 ). The differences notwithstanding, it is well established that pregnancy hyperglycemia increases the risk of adverse events for the mother-child pair, with short- and long-term consequences ( 4 – 6 ). These outcomes could be even worse when GDM is diagnosed in early pregnancy, before 24 weeks of gestation ( 7 , 8 ).

According to the International Diabetes Federation, 21.1 million ( 16 . 7 %) live births among women in 2021 had some form of hyperglycemia in pregnancy, of which 80.3% were due to GDM, 10.6% to diabetes detected prior to pregnancy, and 9.1% to other types of diabetes (including type 1 and type 2) first detected in pregnancy ( 9 ). Affecting approximately 14% of the world's population and up to 18% of Brazilians, GDM is considered a public health problem ( 10 , 11 ). Therefore, many institutions worldwide have emphasized the need to take action to prevent GDM, especially in low- and middle-income countries ( 12 ).

One of the main risk factors for GDM is obesity. Women who become pregnant with a body mass index (BMI) of 30 kg/m^2^ or higher are three to nine times more likely to develop GDM ( 13 ). This is a worrying scenario considering the growing prevalence of obesity in adults, about 13% in the world in 2016 and 20.3% in Brazil in 2019 ( 14 , 15 ). Obesity and GDM are pathophysiologically linked by metainflammation ( 16 ). Pregnant women with previous obesity have a low-grade inflammatory status with elevation of some proinflammatory cytokines, which interferes with insulin signalization, impairing glucose uptake and intensifying insulin resistance during pregnancy ( 17 ).

Both of those conditions are harmful in pregnancy, but each one's adverse outcomes differs among studies, mostly regarding the risk of preeclampsia (PE), cesarean section (CS), a large-for-gestational-age (LGA) newborn or macrosomia, and neonatal intensive care unit (NICU) admission ( 18 – 23 ). For example, although some studies indicate a higher risk of LGA newborns only in patients with obesity-GDM association, others have shown a high risk even among those with isolated obesity ( 19 , 23 ). This disparity may have occurred due to the use of different GDM screening methods (universal *vs.* high-risk population; one-step *vs.* two-step), pregnancy time at BMI measurement (first *vs.* second trimester), and confounding factors, such as high gestational weight gain (GWG).

Therefore, we aimed to evaluate the influence of isolated and associated prepregnancy obesity and GDM on the risk of PE, CS, LGA newborns and NICU admission, adopting the WHO-2013 universal GDM screening and the first trimester BMI assessment ( 2 , 11 , 24 , 25 ).

## MATERIALS AND METHODS

### Study design and ethical standards

A cross-sectional observational study was done from August 1st to December 22nd, 2020, with postpartum women who delivered at a Brazilian public maternity hospital. The study began after approval by the local Research Ethics Committee and the respective Presentation Certificate for Ethical Appreciation (PCEA) – 28786020.5.0000.5363. Following the requirements of Resolution 466/12 from the National Health Council (Brazilian Ministry of Health), which regulates research involving human beings, written informed consent was obtained from all participants.

### Participants and data collection

At the moment of delivery, an interview and an application form were completed by singleton pregnant women age 18 or over without previous DM and with prenatal follow-up at local health basic units – primary care centers in Brazilian's National Health System. Complementary data were collected from prenatal and maternity hospital records. We excluded patients who left the study after signing the informed consent and those with DM first detected in pregnancy (overt diabetes). This last condition was diagnosed in participants if their fasting plasma glucose (FPG) was ≥ 126 mg/dL or 2 h oral glucose tolerance test 75 g (OGTT) was ≥ 200 mg/dL ( 1 , 2 , 11 ).

### Study variables

Based on first trimester BMI (weight and height collected from prenatal records up to 12 weeks of gestation) and GDM screening, patients were allocated to one of four groups: no obesity no GDM (reference group), isolated GDM, isolated obesity, and obesity with GDM. Obesity diagnostic criteria was BMI ≥ 30 kg/m^2^ ( 24 ). GDM was diagnosed as FPG (at any time during the pregnancy) ≥ 92 mg/dL and < 126 mg/dL or one of the following cutoffs in the OGTT 75 g (between 24 and 28 weeks of gestation): FPG ≥ 92 mg/dL and < 126 mg/dL, 1 h ≥ 180 mg/dL, or 2 h ≥ 153 mg/dL and < 200 mg/dL ( 11 ). GDM patients were also treated by the high-risk service of the maternity hospital. Women who did not achieve glycemic target levels after 2 weeks of diet and physical exercises received metformin or insulin (regular and NPH) according to the institution's protocol.

For outcome accounting, we used hospital medical records. Those staff diagnosed PE and LGA newborns based on current standardized definitions our institution adopted, as seen below:

- Preeclampsia (PE): systolic blood pressure at ≥ 140 mmHg and/or diastolic blood pressure at ≥ 90 mmHg on at least two occasions measured four hours apart in previously normotensive women and accompanied by one or more of the following new-onset conditions at or after 20 weeks of gestation: significant proteinuria (≥300 mg at 24 hours or spot urine protein/creatinine ratio ≥ 0.3 mg/mg) or other maternal organ dysfunction (acute kidney injury, liver involvement, neurological complications, hematological complications or uteroplacental dysfunction) ( 26 ).
- Large-for-gestational-age (LGA): birth weight equal to or more than the 90th percentile for a given gestational age (GA) and sex according to INTERGROWTH-21st charts ( 27 ).
- Macrosomia: newborn weighting 4,000 g or over, regardless of the gestational age ( 28 ).

### Statistical analysis

Form contents were scanned in a double entry electronic bank for agreement verification. The statistical analysis was conducted using Statistical Package for the Social Sciences software (SPSS, IBM Corp., Armonk, NY, US), version 26.

Quantitative variables were presented as means and standard deviation and qualitative ones as absolute and relative frequencies. We verified the equality hypothesis between group averages using the t-test or Mann-Whitney test, variables distribution using the Kolmogorov-Smirnov test, and group homogeneity using the Chi-square test or Fisher's exact test for frequencies below 5.

The relationship between obesity and GDM with adverse outcomes were explored using multivariate logistic regression models and the variables’ effect was estimated by odds ratio (OR) adjusted for confounding factors, with a 95% confidence interval (CI). The confounding factors adopted were patient's age, GWG, GA at delivery, parity, previous CS, and smoking. We considered *P* values < 0.05 statistically significant.

## RESULTS

### Sample analysis

**Figure 1:**
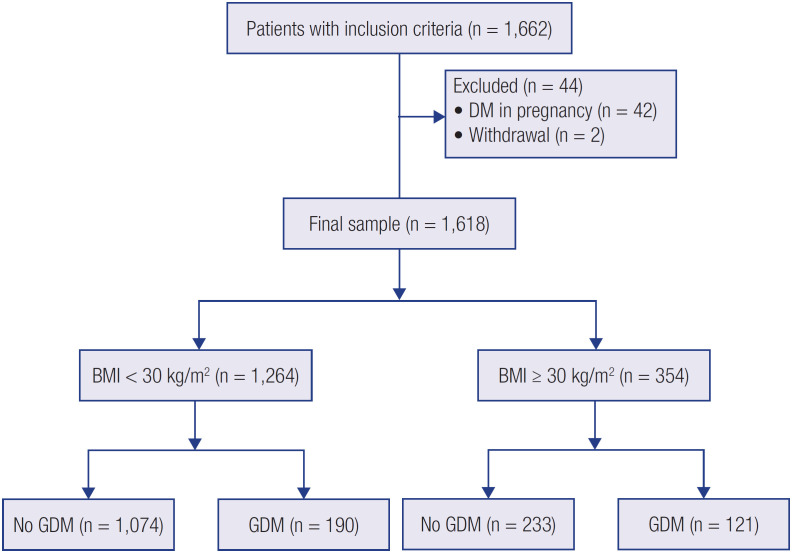
Flowchart of sample composition.

BMI: body mass index; DM: diabetes mellitus; GDM: gestational diabetes mellitus.

Source: the authors.

During the study period, 2,284 pregnant women delivered at our institution, but 622 of them were not included due to absent prenatal records of first trimester BMI and GDM screening. Of the 1,662 patients who met all the inclusion criteria we excluded 44 participants, 42 due to DM diagnosed in pregnancy and 2 due to withdrawal. The final sample of 1,618 participants were stratified in the following groups: no obesity no GDM (66.38%), isolated GDM (11.74%), isolated obesity (14.40%), and obesity with GDM (7.48%) ( Figure 1 ).

Most of these women were between 20 and 29 years old, white, high schooling, multiparous and without prepregnancy obesity. The mean pregestational BMI was 26.09 kg/m^2^ and 21.88% had obesity. GDM counts for 19.22% and half of them required pharmacotherapy, but the majority used only metformin ( Table 1 ).

**Table 1:** Studied population profile

Maternal characteristics		
Age (years)		
	≥18 e <20	121 (7.48%)
	20-24	500 (30.90%)
	25-29	443 (27.38%)
	30-34	326 (20.15%)
	35-39	172 (10.63%)
	40-44	49 (3.03%)
	≥45	7 (0.43%)
Ethnicity		
	White	1,309 (80.90%)
	Black	66 (4.08%)
	Other	243 (15.02%)
Schooling		
	Elementary school	403 (24.90%)
	High school	1002 (61.93%)
	Higher education	213 (13.16%)
Prepregnancy BMI		
	<30 kg/m^2^	1,264 (78.12%)
	≥30 kg/m^2^	354 (21.88%)
Smoking		122 (7.54%)
C. hypertension		99 (6.11%)
Parity		
	Primiparous	511 (31.58%)
	Multiparous	1,107 (68.42%)
Obstetric history		
	CS	414 (25.58%)
	Macrosomia	147 (9.09%)
GDM		311 (19.22%)
GDM treatment		
	Diet only	152 (48.88%)
	Diet and metformin	121 (38.90%)
	Diet and insulin	20 (6.43%)
	Diet, metformin and insulin	18 (5.79%)
PE		146 (9.02%)
Delivery GA		
	<37 weeks	118 (7.29%)
	≥37 weeks	1,500 (92.71%)
Delivery mode		
	Vaginal	923 (57.04%)
	Forceps	14 (0.86%)
	CS	681 (42.08%)
Emergency CS		289 (17.86%)
**Newborn characteristics**		
Birth weight		
	<1,500 g	20 (1.24%)
	1,500 g-2,499 g	77 (4.76%)
	2,500 g-3,999 g	1,400 (86.53%)
	≥4,000 g	121 (7.48%)
Classification		
	SGA	128 (7.91%)
	AGA	1,256 (77.62%)
	LGA	234 (14.46%)
NICU admission		25 (7.72%)

Data in numbers (percentage). BMI: body mass index; C. hypertension: chronic hypertension; Primiparous: women with no previous childbirths; Multiparous: women with one or more previous childbirths; CS: cesarean section; Macrosomia: newborn weighting 4,000 g or over; GDM: gestational diabetes mellitus; PE: preeclampsia; GA: gestational age; Classification: birth weight by GA and sex; SGA: small-for-gestational-age; AGA: appropriate-for-gestational-age; LGA: large-for-gestational-age. NICU: neonatal intensive care unit.

The mean GA at delivery was 38 weeks and 5 days, with less than 8% preterm births. The newborns’ mean weight was 3,291 g, with 14.46% being LGA and half of those being macrosomic. NICU admission was needed for 7.72% of the newborns ( Table 1 ).

Vaginal delivery was the leading birth mode (57.04%) followed by CS (42.08%). Most CSs were non-emergency procedures and resulted from previous CSs (two or more) and macrosomia reported on ultrasound. When emergency CSs were needed, the most common indications were failure of labor progression, non-reassuring fetal situation, and PE with maternal or fetal instability.

### Analysis by group

Comparing the groups’ profiles, the participants’ mean age was similar between groups as it was the gestational mean age at delivery. Of the three studied groups, the isolated-GDM group had the highest mean GWG (13.7 kg) but the lowest mean BMI (24.7 kg/m^2^) and previous CS rate (28.4%), whereas the isolated-obesity group had the highest previous CS rate (35.2%). Last, the obesity-GDM group had the highest mean BMI (35.5 kg/m^2^) and the lowest mean GWG (8.0 kg) ( Table 2 ).

**Table 2:** Characteristics and outcomes by group

	No obesity		Obesity		*P* value
	No GDM*	GDM	No GDM	GDM	
	n = 1,074 (66.38%)	n = 190 (11.74%)	n = 233 (14.40%)	n = 121 (7.48%)	
**Characteristics**					
Age (years)^a^	26.4 (±5.9)	29.2 (±6.2)	28.2 (±5.4)	29.9 (±5.8)	0.000
BMI (kg/m^2^)^a^	23.5 (±3.3)	24.7 (±3.3)	33.7 (±3.7)	35.5 (±4.8)	0.000
GWG (kg)^a^	13.8 (±6.9)	13.7 (±6.8)	10.0 (±6.9)	8.0 (±7.3)	0.000
Smoking^b^	78 (7.3%)	15 (7.9%)	22 (9.4%)	7 (5.8%)	0.594
Metformin/Insulin^b^	-	134 (70.6%)	-	93 (76.9%)	0.002
C. hypertension^b^	34 (3.2%)	14 (7.4%)	21 (9%)	30 (24.8%)	0.000
Primiparous^b^	377 (35.1%)	45 (23.7%)	62 (26.6%)	27 (22.3%)	0.001
Multiparous^b^	697 (64.9%)	145 (76.3%)	171 (73.4%)	94 (77.7%)	0.001
Previous CS^b^	237 (22.1%)	54 (28.4%)	82 (35.2%)	41 (33.9%)	0.000
Delivery GA (weeks)^a^	38.8 (±1.9)	38.2 (±1.4)	38.8 (±2.1)	38.0 (±1.8)	0.000
**Outcomes**					
PE^b^	71 (6.6%)	18 (9.5%)	35 (15%)	22 (18.2%)	0.000
CS^b^	411 (38.3%)	86 (45.3%)	116 (49.8%)	68 (56.2%)	0.000
Emergency CS^b^	195 (18.2%)	26 (13.7%)	42 (18.0%)	26 (21.5%)	0.332
Newborn weight (g)^a^	3280 (±544)	3270 (±511)	3340 (±568)	3329 (±540)	0.184
LGA newborn^b^	134 (12.5%)	32 (16.8%)	43 (18.5%)	25 (20.7%)	0.028
Preterm newborn^b^	77 (7.2%)	15 (7.8%)	18 (7.7%)	8 (6.6%)	0.039
NICU admission^b^	69 (6.4%)	23 (12.1%)	20 (8.6%)	13 (10.7%)	0.024

Data in mean and standard deviation (a) or numbers and percentage (b); GDM: gestational diabetes mellitus; BMI: body mass index; GWG: gestational weight gain; C. hypertension: chronic hypertension; GA: gestational age; PE: preeclampsia; CS: cesarean section; LGA: large-for-gestational-age; Preterm newborn: with less than 37 weeks; NICU: neonatal intensive care unit; *P* values compared with reference group – no obesity no GDM (*), adjusted for maternal age, GWG, GA at delivery, parity, previous CS and smoking.

Concerning the adverse outcomes, the incidence of PE, CS, LGA newborns and NICU admission increased in all studied groups compared to the reference one. However, after we adjusted for confounding factors and considering the statistical significance, these events’ likelihood differ among groups. As a result, patients with isolated obesity had a high chance of PE (OR = 2.162; *P* = 0.001), patients with isolated GDM had a high chance of CS (OR = 1.736; *P* = 0.011) and NICU admission (OR = 2.322; *P* = 0.007), and those with obesity-GDM association had a high chance of PE (OR = 1.934; *P* = 0.028), CS (OR = 1.925; *P* = 0.017) and LGA newborns (OR = 1.815; *P* = 0.040) ( Table 3 ).

**Table 3:** Chance of adverse perinatal outcomes by group

		n cases/group	*P* value	OR	95% CI
PE					
	Isolated GDM	18/190	0.335	1.328	0.746-2.363
	Isolated obesity	35/233	0.001	2.162	1.364-3.426
	Obesity with GDM	22/121	0.028	1.934	1.074-3.484
CS					
	Isolated GDM	86/190	0.011	1.736	1.136-2.652
	Isolated obesity	116/233	0.056	1.473	0.990-2.190
	Obesity with GDM	68/121	0.017	1.925	1.124-3.298
LGA newborn					
	Isolated GDM	32/190	0.383	1.246	0.760-2.042
	Isolated obesity	43/233	0.183	1.350	0.868-2.100
	Obesity with GDM	25/121	0.040	1.815	1.027-3.204
NICU admission					
	Isolated GDM	23/190	0.007	2.322	1.265-4.261
	Isolated obesity	20/233	0.321	1.370	0.735-2.555
	Obesity with GDM	13/121	0.332	1.482	0.669-3.280

PE: preeclampsia; CS: cesarean section; LGA: large-for-gestational-age; NICU: neonatal intensive care unit; GDM: gestational diabetes mellitus; OR: odds ratio; 95% CI: confidence interval of 95%; *P* values and OR compared with reference group (no obesity no GDM), adjusted for maternal age, GWG, GA at delivery, parity, previous CS and smoking; *P* value < 0.05 statistically significant.

## DISCUSSION

In this study, we analyzed the influence of obesity, GDM, and their association in adverse perinatal outcomes adopting a different methodology, which contributed to its strength. First, unlike studies in which the researchers calculated BMI based on weight self-reported or measured at the second trimester, we obtained it using anthropometric data recorded in the first trimester. That method reduced the power of GWG as a confounding factor, reinforced by its inclusion in adjustable OR calculation ( 29 , 30 ). Second, whereas in many studies GDM was measured only in the second trimester or in high-risk groups, our sample was universally screened for GDM since the first trimester. This allowed the exclusion of patients with overt diabetes, reduced loss of GDM cases in low-risk populations and optimized early GDM detection and management, enhancing the measurement of the impact of treated GDM on pregnancy outcomes ( 7 , 12 ).

The prevalence of obesity (21.88%) was lower than Brazilian's average (29.50%), probably because most participants were under 25 years old, which is the national age group with the lowest obesity rate ( 15 ). Meanwhile, the prevalence of GDM (19.22%) was compatible with Brazil's (18%) and higher in women with obesity, confirming high BMI's impact on GDM development ( 11 , 30 ).

Prepregnancy obesity was the main factor that increased the chance of PE, even after being adjusted for chronic hypertension and excessive GWG, possibly due to the interference of obesity in pregnancy vascular adaptation, characterized by leptin (proinflammatory) and endothelin (vasoconstrictor) increases ( 31 ). This is in line with the findings of Weschenfelder and cols., who used the same GDM screening, considered GWG, and reported no independent effect of GDM on the risk of PE. They also found that high BMI was the major impacting factor ( 22 ).

GDM was the principal condition related to the chance of CS, despite some authors attributing this risk mainly to obesity and considering GDM only an amplifier. This divergence may be related to different obesity and CS rates in those populations compared to the Brazilian rates ( 32 , 33 ). In the Saudi study by Wahabi and cols., 44% of women had obesity ( *vs.* 21.88% in our study), and in the Finland study by Ijäs and cols., only 18.07% of women had CS ( *vs.* 42.08% in our study) ( 19 , 21 ). In addition to that, the first study used BMI of the second trimester, and in neither study GWG was considered, which could have enhanced the risk of adverse outcomes attributed to obesity.

Like other authors, we noted an increased chance of LGA newborns in mothers with obesity, GDM, and their combination, but in our study this chance reached statistical significance only in patients with obesity-GDM association. This is probably related to a higher prepregnancy BMI and increased need for GDM drug therapy in this group, which is likely a result of hyperinsulinemia and dyslipidemia in these patients, with more glucose and lipids crossing the placental barrier and contributing to fetal adiposity ( 34 ). Unfortunately, detailed data about group differences in mean BMI and GDM treatment were not presented in those previously cited studies, limiting the comparison ( 19 , 21 ).

Similar to Weschenfelder and cols., we found that isolated GDM increased the chance of NICU admission more than twofold ( 22 ). This reflects the power of GDM even in pregnant women without risk factors, and the limitation of its treatment to prevent this adverse outcome ( 23 ). Interestingly, unlike in other studies, the chance of NICU admission was not significantly higher in the obesity-GDM group, possibly due to this group's lower rate of preterm newborns or even the limited number of participants. This highlights the importance of more studies to confirm this relationship.

Despite this study's aforementioned strength limitations were inevitable, such as sample size, which may have hindered OR power and generalizability. The more extensive use of metformin than of insulin might have interfered with the results due to differences in glycemic control, but with lower impact between groups considering their similar proportion of use ( 35 ). Last, the coronavirus pandemic (COVID-19) impacted data collection as it affected people's mobility by reducing bus fleet and income, leading to delays in diagnosis and treatments performed at prenatal care follow-up ( 36 ).

In conclusion, isolated maternal obesity and GDM increase the chance of various adverse perinatal outcomes. These conditions jointly increase the chance of more unfavorable events, highlighting the importance of prepregnancy obesity in perinatal prognosis and encouraging its treatment before conception. As some GDM risks are still present besides its treatment, actions to prevent hyperglycemia in pregnancy are needed. When prevention is not achieved, pregnant women with obesity-GDM association should be followed closely.
